# Supplementary material for: Parkinsonism Sac domain mutation in Synaptojanin-1 affects ciliary properties in iPSC-derived dopaminergic neurons
Source: Proc Natl Acad Sci U S A. 2024 Apr 18;121(17):e2318943121. doi: 10.1073/pnas.2318943121 (PMC11047088; doi:10.1073/pnas.2318943121)
Supplement: Supplementary file 1 — Appendix 01 (PDF) [file pnas.2318943121.sapp.pdf]

Supplementary Figure 1

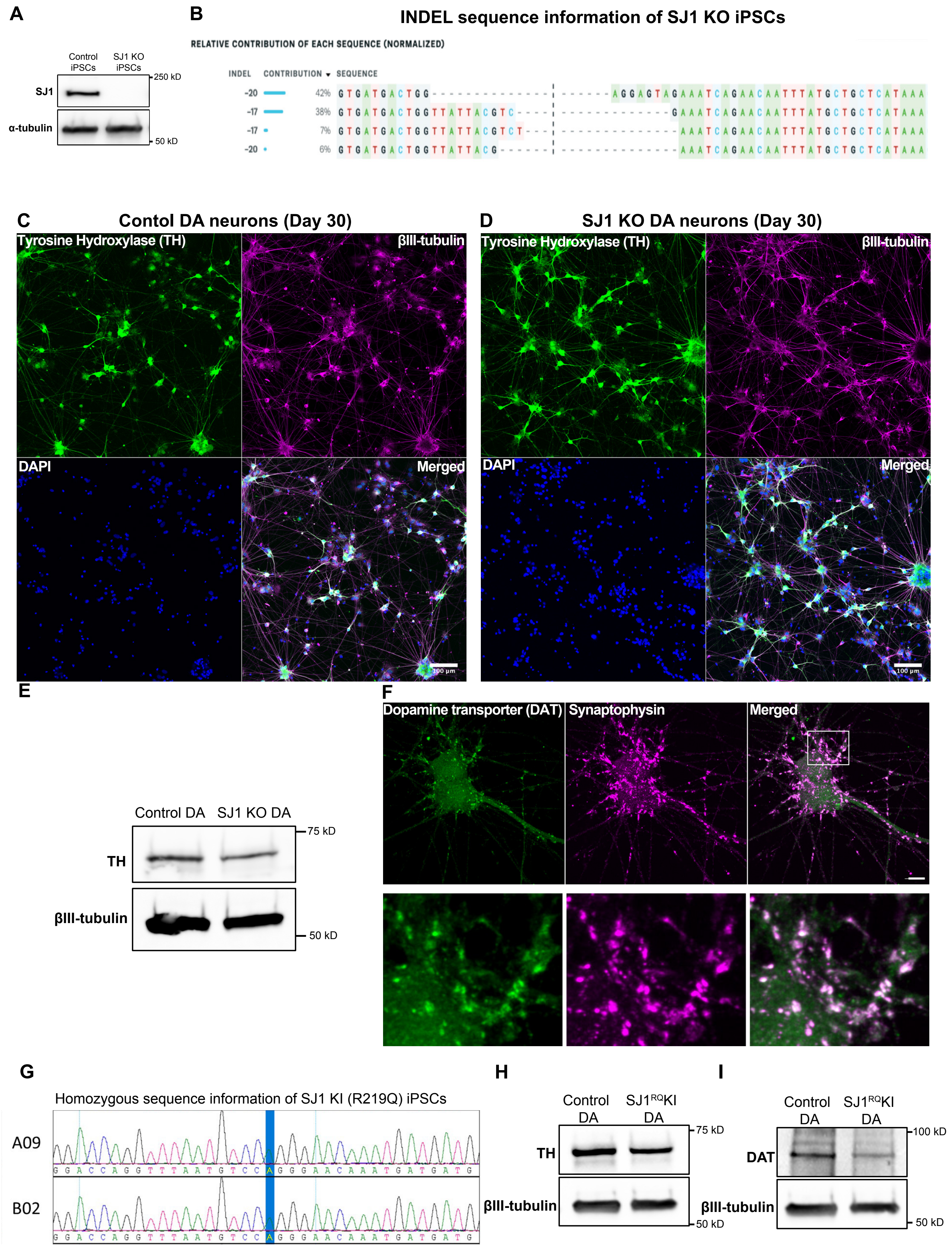

Supplementary Figure 1: Genetic and biochemical validation of SJ1 KO and SJ1^{RQ}KI iPSCs and iPSC-derived DA neurons

(A) Anti-SJ1 and anti-α-tubulin (loading control) western blot of control and edited WTC11 iPSCs. (B) Normalized relative contribution of INDEL sequences (normalized) of SJ1 KO iPSCs. (C and D) Fluorescence images of control and SJ1 KO iPSC-derived DA neurons (day 30) immunolabeled with antibodies directed against tyrosine hydroxylase (TH) (green) and βIII-tubulin (magenta). Nuclei were labeled with DAPI (blue)(Scale bars, 100 µm). (E) Anti-TH and anti-βIII-tubulin (loading control) western blot of control and SJ1 KO DA neurons (day 30). (F) Fluorescence images of control iPSC-derived DA neurons (day 30) immunolabeled with antibodies directed against dopamine transporter (DAT) (green) and synaptophysin (magenta). (G) Chromatogram sequences of two homozygous KOLF2.1 SJ1^{RQ}KI iPSC clones. (H) Anti-TH and anti-α-tubulin (loading control) western blot of control and edited KOLF2.1 iPSCs. (I) Anti-DAT and anti-βIII-tubulin (loading control) western blot of control and edited KOLF2.1 iPSCs.

Supplementary Figure 2

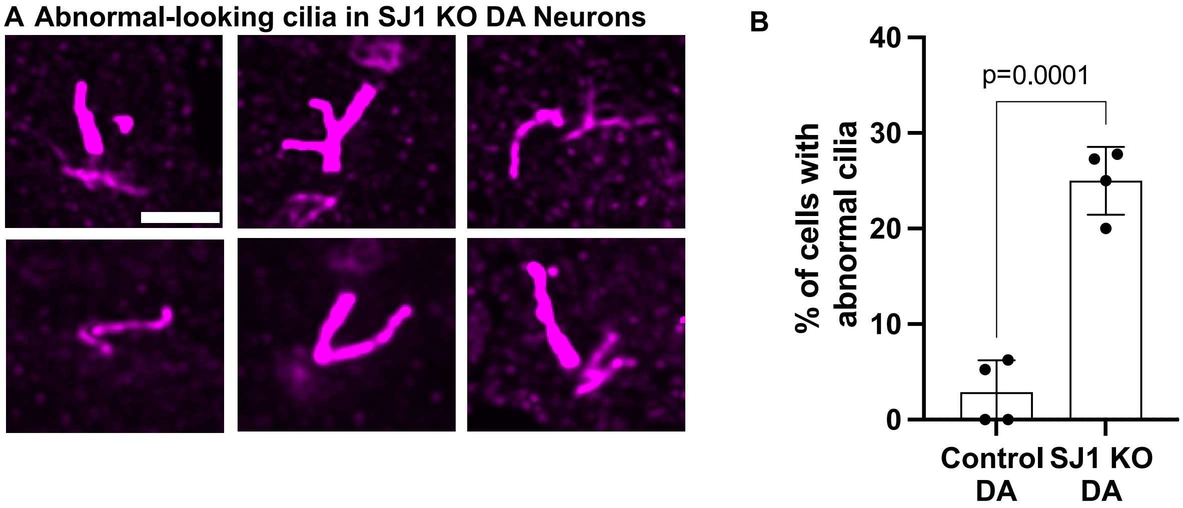

Supplementary Figure 2: SJ1 KO DA neurons show abnormal ciliary morphology

(A) Gallery of fluorescence images of Arl13b-labeled abnormal cilia from SJ1 KO iPSC-derived DA neurons. Scale bar, 2 µm. (B) Percentage of cells with abnormal cilia in control and SJ1 KO DA neurons (day 30) represented as mean ± S.E.M. (from four independent experiments; n ≥ 10 cells per experiment).

Supplementary Figure 3

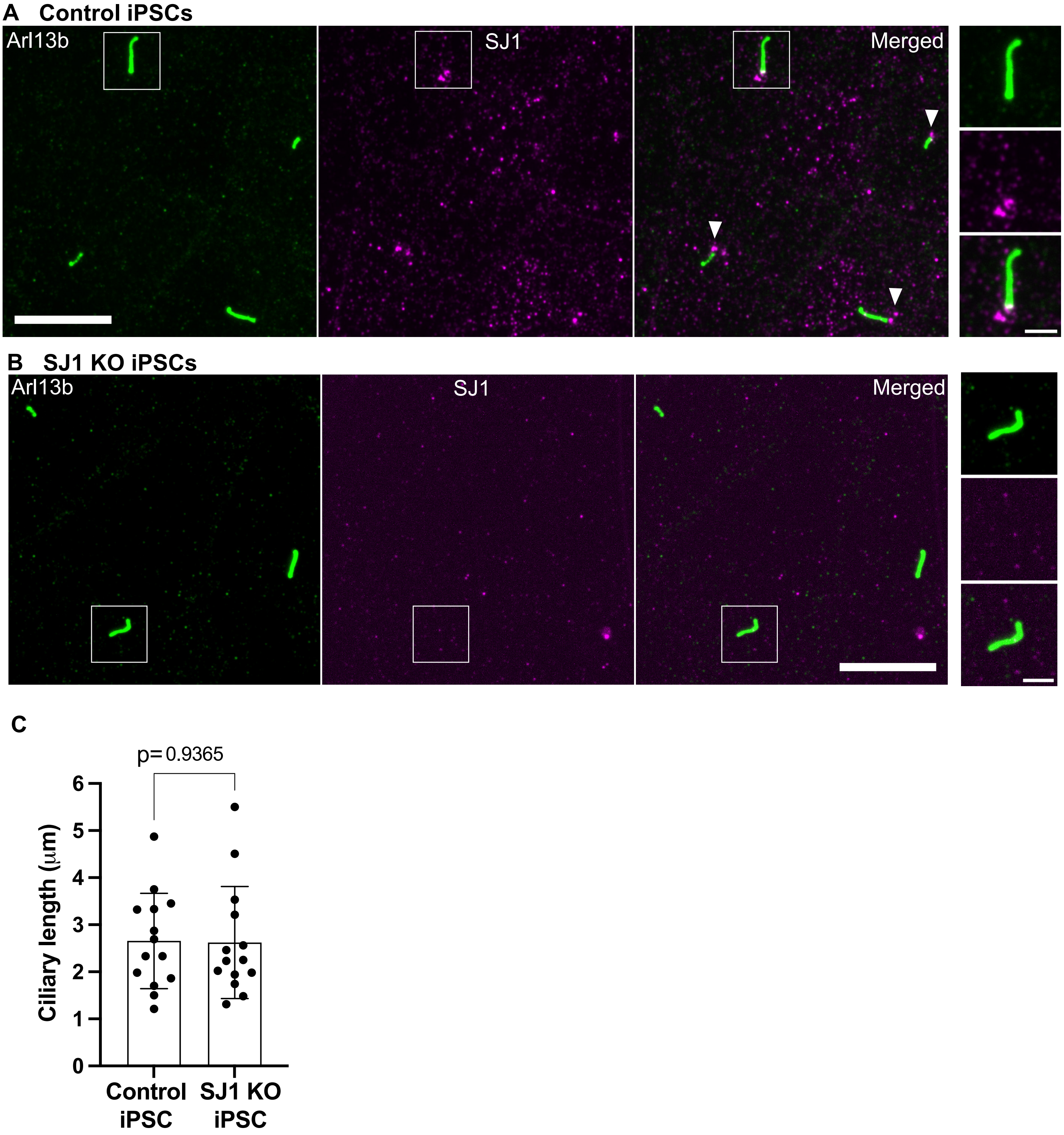

Supplementary Figure 3: No change in ciliary length of control and SJ1 KO iPSCs

Fluorescence image of Control (A) and SJ1 KO (B) iPSCs immunolabeled with antibodies against Arl13b (green) and SJ1 (magenta) showing no change in ciliary length. High magnifications of boxed areas in (A, B) are shown at right. (Scale bars, 10 µm; cropped areas: 2 µm). (C) Quantification of cilia length in control and SJ1 KO iPSCs represented as mean ± S.D. (data pooled from two independent experiments; n ≥ cells per experiment).

Supplementary Figure 4

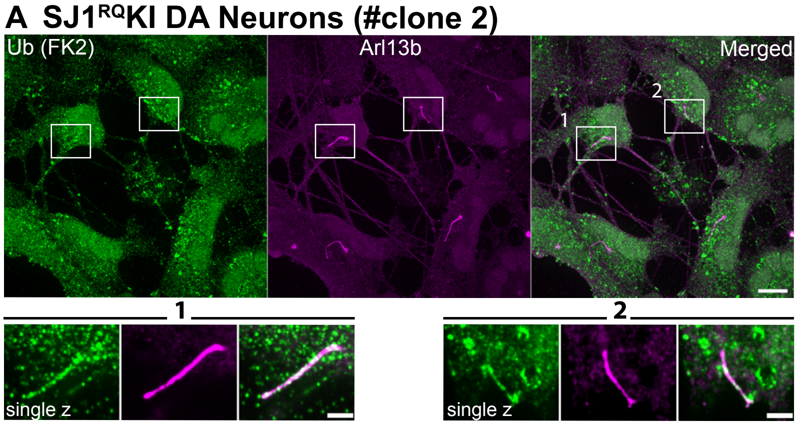

Supplementary Figure 4: Ubiquitination in SJ1^{RQ}KI DA neurons

(A) Fluorescence image of SJ1^{RQ}KI (B) DA neurons clone #2 immunolabeled with FK2 antibodies directed against lysine 63-linked ubiquitin chains (green) and against Arl13b (magenta). High magnifications of the boxed areas are shown below the main panels. (Scale bars, 10 µm; cropped areas: 2 µm).

Movie S1: Live fluorescence imaging of calcium dynamics in control DA neurons (white) labeled with Fluo-4 dye show numerous spontaneous spikes. The frames were recorded at 500-millisecond intervals over a period of 1 minute. Display rate is 10 frames/sec. Scale bar, 10 µm.

Movie S2: Live fluorescence imaging of calcium dynamics in $SJ1^{RQ}KI$ DA neurons (white) labeled with Fluo-4 dye show significant reduction in calcium spikes when compared to control. The frames were recorded at 500-millisecond intervals over a period of 1 minute. Display rate is 10 frames/sec. Scale bar, 10 µm.

Table S1. List of antibodies/dyes used in this study.

Target Protein	Company; Catalog number; RRID	Antibody species	Working dilution for immunofluorescence	Working dilution for immunoblotting
βIII-tubulin (TUJ1)	BioLegend; 801201 (RRID:AB_2728521)	Mouse	1:500	1:3000
βIII-tubulin	Abcam; ab18207 (RRID:AB_444319)	Rabbit	1:500	N/A
α-Tubulin	Sigma Aldrich; T5168 (RRID:AB_477579)	Mouse	N/A	1:10000
Tyrosine Hydroxylase	EMD Millipore; 657012 (RRID:AB_2313810)	Rabbit	1:300	N/A
Acetylated-tubulin	Sigma-Aldrich; T6793 (RRID:AB_477585)	Mouse	1:500	N/A
ARL13b	NeuroMab; N295B/66 75-287 (RRID:AB_2877361)	Mouse	1:250	N/A
ARL13b	Proteintech;17711-1-AP (RRID:AB_2060867)	Rabbit	1:250	N/A
γ-tubulin	Sigma-Aldrich; T6557 (RRID:AB_477584)	Mouse	1:500	N/A
Adenylate Cyclase III	Abcam; 125093 (RRID:AB_10975307)	Rabbit	1:200	N/A
Synaptojanin-1	Novus Biologicals; NBP1-87842 (RRID:AB_11047653)	Rabbit	1:250	1:1000
Amphiphysin-2	Sigma-Aldrich; 99D (RRID:AB_258663)	Mouse	1:250	N/A
Clathrin light chain	EMD Millipore; AB9884 (RRID:AB_11211734)	Rabbit	1:250	N/A
Auxilin	De Camilli Lab (RRID:AB_2878951)	Rabbit	1:250	N/A
Ub (FK1)	EMD Millipore; 04-262 (RRID:AB_612094)	Mouse	1:500	1:1000
Ub (FK2)	Enzo Life Sciences; BML-PW8810 (RRID:AB_10541840)	Mouse	1:500	1:1000
$Ca_v1.3$	Alomone labs;ACC-005	Rabbit	1:250	N/A

	(RRID:AB_2039775)			
DAPI	Thermo Fischer Scientific; D3571	N/A	1:1000 (stock: 1 mg/ml)	N/A
Synapsin I	Synaptic Systems; 106103 (RRID:AB_11042000)	Rabbit	1:250	N/A
Synaptophysin	Synaptic Systems; 101002 (RRID:AB_887905)	Rabbit	1:500	N/A
Synaptophysin	Synaptic Systems; 101001 (RRID:AB_2617061)	Mouse	1:250	N/A
Dopamine transporter	EMD Millipore; MAB369 (RRID:AB_2190413)	Rat	1:500	1:1000
Vinculin	Sigma-Aldrich; V4505 (RRID:AB_477617)	Mouse	N/A	1:1000
Fluo-4	Thermo Fischer Scientific; F14201	N/A	1:1000 (stock: 1 mM)	N/A
